# Chromatin Regulators Ahc1p and Eaf3p Positively Influence Nitrogen Metabolism in *Saccharomyces cerevisiae*

**DOI:** 10.3389/fmicb.2022.883934

**Published:** 2022-05-10

**Authors:** Yu Chen, Weizhu Zeng, Wenjian Ma, Wei Ma, Jingwen Zhou

**Affiliations:** ^1^Science Center for Future Foods, Jiangnan University, Wuxi, China; ^2^Key Laboratory of Industrial Biotechnology, Ministry of Education and School of Biotechnology, Jiangnan University, Wuxi, China; ^3^Engineering Research Center of Ministry of Education on Food Synthetic Biotechnology, Jiangnan University, Wuxi, China; ^4^Jiangsu Province Engineering Research Center of Food Synthetic Biotechnology, Jiangnan University, Wuxi, China

**Keywords:** amino acid utilization, nitrogen preference, Ahc1p, Eaf3p, histone acetylation

## Abstract

There is a complex regulatory network of nitrogen metabolism in *Saccharomyces cerevisiae*, and many details of this regulatory network have not been revealed. This study explored the global regulation of nitrogen metabolism in *S. cerevisiae* from an epigenetic perspective. Comparative transcriptome analysis of *S. cerevisiae* S288C treated with 30 nitrogen sources identified nine chromatin regulators (CRs) that responded significantly to different nitrogen sources. Functional analysis showed that among the CRs identified, Ahc1p and Eaf3p promoted the utilization of non-preferred nitrogen sources through global regulation of nitrogen metabolism. Ahc1p regulated nitrogen metabolism through amino acid transport, nitrogen catabolism repression (NCR), and the Ssy1p-Ptr3p-Ssy5p signaling sensor system. Eaf3p regulated nitrogen metabolism via amino acid transport and NCR. The regulatory mechanisms of the effects of Ahc1p and Eaf3p on nitrogen metabolism depended on the function of their histone acetyltransferase complex ADA and NuA4. These epigenetic findings provided new insights for a deeper understanding of the nitrogen metabolism regulatory network in *S. cerevisiae*.

## Introduction

Nitrogen metabolism plays an essential role in the growth and fermentation of *Saccharomyces cerevisiae*. There are complex regulatory mechanisms for nitrogen utilization in this yeast, including sensing, transport, and catabolism (Zhang et al., 2018b). Nitrogen catabolism repression (NCR) is one of the most important upstream regulatory mechanisms controlling nitrogen metabolism in *S. cerevisiae* (Nair and Sarma, 2021). Genes regulated by NCR transcription factors in *S. cerevisiae* are referred as NCR genes (Zhang et al., 2018b). The NCR pathway regulates the expression of NCR genes through NCR transcription factors, causing cells to exhaust good nitrogen sources before using poor sources (Magasanik and Kaiser, 2002). As a result, nitrogen sources are not used efficiently, resulting in the wastage of nitrogen sources and limited metabolic processes in cells (Zhang et al., 2018b).

Previous studies have shown that NCR transcription factors are regulated by target of rapamycin complex 1 (TORC1; Molinet et al., 2019). However, TORC1 is not the only upstream regulatory pathway for NCR transcription factors (Fayyadkazan et al., 2014); NADP-glutamate dehydrogenase activity of Gdh1 (Fayyad-Kazan et al., 2016) and general amino acid control (GAAC; Tate et al., 2017) also have important regulatory effects on NCR. In addition, there may be unknown regulatory mechanisms that regulate NCR transcription factors or cooperate with NCR transcription factors to regulate NCR-sensitive genes. Our previous study found that the nucleosome abundance of *S. cerevisiae* S288C was significantly affected by the nitrogen source preference (Zhang et al., 2016). Because nucleosome changes are an important form of chromatin regulation (McKittrick et al., 2004; Mobius et al., 2013), chromatin regulation may be related to NCR in *S. cerevisiae*.

Eukaryotic genome transcription is regulated mainly by chromatin, and a series of chromatin regulators (CRs) with different functions cooperate to regulate chromosome activity and thereby control gene transcription (Morrison, 2020). In addition to the arrangement of nucleosomes, histone modification is another important form of chromatin regulation (Saha, 2020; Reyes et al., 2021), in which histone acetylation regulation is an essential mechanism of gene expression regulation (Kassem et al., 2017). Acetylation of histones converts chromatin from a compact to an active state, favoring gene activation (Culbertson and Shogren-Knaak, 2021). The Ada2-Ada3-Gcn5-Ahc1 (ADA) acetyltransferase complex is a histone acetyltransferase in eukaryotes that preferentially acetylates 14 lysine residues of H3 (Helmlinger et al., 2021). Ahc1p (Ada Histone acetyltransferase complex Component) is a unique subunit of the ADA complex that is essential for its structure and function (Culbertson and Shogren-Knaak, 2021). In addition, nucleosome acetyltransferase of H4 (NuA4) is also a critical acetyltransferase complex in eukaryotes (Sathianathan et al., 2016), and its subunit Eaf3p (Esa1p-Associated Factor) is also a subunit of the deacetylase complex Rpd3S (Biswas et al., 2008; Okuda and Nishimura, 2020; Lee et al., 2021). Eaf3p identifies the corresponding histone methylation sites through the N-terminal chromodomain of the nucleosome in the gene-coding region to locate NuA4 and Rpd3S (Ruan et al., 2016). However, the roles of Ahc1p and Eaf3p in nitrogen metabolism have not been reported.

Research on the regulatory mechanism of nitrogen metabolism in *S. cerevisiae* has been ongoing in recent years, but the complete regulatory network has not been fully elucidated. Herein, we discovered a correlation between CRs and nitrogen metabolism through global transcriptomic analysis under the short-term impact of different nitrogen sources. Subsequent functional analysis revealed that the CRs Ahc1p and Eaf3p promoted the utilization of non-preferred nitrogen sources by simultaneously regulating multiple nitrogen metabolism-related pathways. Two key chromatin regulators, Ahc1p and Eaf3p, were found to exert a global regulatory effect on nitrogen metabolism in *S. cerevisiae* S288C, and these effects were found to depend on the function of their histone acetyltransferase complexes ADA and NuA4. The findings helped to elucidate the regulatory network controlling nitrogen metabolism in *S. cerevisiae*.

## Materials and Methods

### Strains and Media

All strains and plasmids used are listed in Supplementary Tables S1 **,** S2. *S. cerevisiae* S288C (Daran-Lapujade et al., 2003) was used as control strain. Yeast strains were activated in yeast extract peptone dextrose medium (YPD, 10 g/l yeast extract, 20 g/l peptone, 20 g/l glucose) and cultured in yeast nitrogen base medium (YNB, without ammonia and amino acids) containing 2% (*w*/*v*) glucose and 0.05 g/l amino acids at 30°C with shaking at 220 rpm. Agar (2%) was added to the medium when necessary. Nitrogen sources were added to the stock medium to a final concentration of 10 mM (5 mM for tryptophan).

The *ura3* deletion mutant strain S288C-Δ*ura3* was constructed by homologous recombination and screened using yeast nitrogen base medium with 5-fluoroorotic acid monohydrate (5-FOA) and uracil. S288C-Δ*ura3* was used as the host strain for gene knockout and overexpression. The CRISPR-Cas9 plasmid pRS426-Cas9-sgRNA (Supplementary Figure S1) was used for gene knockout and the multiple-copy plasmid pY26-TEF-GPD (Li et al., 2008) was used for gene overexpression. Overexpression plasmids were constructed using a Clonexpress II One-step Cloning Kit (Vazyme, Nanjing, China). The engineered plasmid was confirmed by DNA sequencing, transformed into the corresponding strain using the lithium acetate method (Gietz and Schiestl, 2007), and positive transformants were screened by colony PCR and verified by DNA sequencing.

### Determination of Sample Processing Time

In order to determine the impact time point with obvious response to nitrogen source but little effect on yeast cell growth, three different nitrogen sources, preferred glutamine, arginine, and non-preferred urea were used for pre-detection. Yeast cells growing to the logarithmic metaphase were treated in three nitrogen sources for 1 h, 2 h, and 3 h, respectively. Nitrogen metabolism-related genes *GLN3* (GLutamiNe metabolism), *GZF3* (Gata Zinc Finger protein), *TOR1* (Target Of Rapamycin), and growth-related genes *RAP1* (Repressor/Activator site binding Protein 1) and *PFK1* (PhosphoFructoKinase) were selected for real-time quantitative PCR analysis.

### Sample Preparation and RNA Sequencing

A single colony of S288C was inoculated in YPD liquid medium for the first activation and after 12 h of culture was transferred to YPD liquid medium for the second activation at an initial concentration of OD_600_ = 0.2. The seed solution was cultured for 6 h to the logarithmic period (OD_600_ = 1.2), then centrifuged and washed with phosphate-buffered saline (PBS) after activation. Next, 10 ml of single nitrogen source medium was added to ammonium sulfate-free YNB medium with a final concentration of 10 mM (5 mM for tryptophan; Supplementary Table S3). Cells were harvested after short-term culture and immediately frozen in liquid nitrogen. RNA was extracted using a HiPure Yeast RNA kit (Magen, Guangzhou, China) following the manufacturer’s protocol. Three independent replicates were carried out for each treatment and RNA-Seq experiment.

Sequencing libraries were generated using an NEBNext Ultra RNA Library Prep Kit for Illumina (NEB, United States) following the manufacturer’s recommendations, and sequenced on an Illumina HiSeq platform. Clean data obtained by removing reads containing adapter, ploy-N, and low-quality reads from raw data were used for subsequent analysis. Hisat2 v2.0.5 was used for building the index of the reference genome and aligning paired-end clean reads to the reference genome. Novel transcript assembly was performed using StringTie software, followed by database annotation of novel transcripts, such as Pfam, GO, and KEGG. Novogene Bioinformatics Technology Co. Ltd. analyzed the RNA-Seq libraries, and gene transcription levels were calculated according to the fragments per kilobase of exon per million mapped reads (FPKM) method. The DESeq2 R package (1.16.1) was used to analyze differences in transcription levels between groups, and genes with an adjusted *value of p* <0.05 and |log_2_foldchange| >0.0 were assigned as differentially expressed. Urea was used as a control to analyze gene expression changes of *S. cerevisiae* S288C under different nitrogen source conditions.

### Enrichment and Clustering of Differentially Expressed Genes

MultiExperiment Viewer (MeV) software (Howe et al., 2011) was used for enrichment analysis and hierarchical clustering of differentially expressed genes (DEGs). DEGs were clustered using log2FoldChange as the criterion, the complete linkage method, and the average dot size as a measure of the distance between gene transcription profiles. The Kyoto Encyclopedia of Genes and Genomes (KEGG) database was used to explore the functions of biological systems (cells, organisms, and ecosystems) based on information at the molecular level in molecular data sets generated by high-throughput experimental technologies.^1^ The ClusterProfiler R package was used to detect the statistical enrichment of differentially genes in KEGG pathways.

### Screening and Clustering of Differential NCR Genes and CRs

NCR genes (Godard et al., 2007) and CRs (Venters et al., 2011) with expression changes in all DEGs were manually selected based on previous reports. MeV software was used for hierarchical clustering analysis of differential NCR genes and CRs. The distribution of differential NCR genes and CRs among the DEGs was displayed by the Venn diagram.

### Culture and Growth Status Determination of Strains

Activation of yeast cells was carried out in YPD medium and cells were cultured to the mid-logarithmic phase. The seed liquid was transferred to YNB medium supplemented with corresponding amino acids for further culture, and the initial OD_600_ (Amberg et al., 2006) was 0.1. Recombinant strains were inoculated in YNB liquid medium with three different amino acids (Glutamine, arginine, and proline at a final concentration of 100 mM) as the sole nitrogen source, and fermented at 30°C with shaking at 220 rpm for 48 h. The OD_600_ was measured every 3 h to calculate the maximum growth rate (μ_max_).

### High-Performance Liquid Chromatography Analysis

The amount of urea accumulation was determined by examining the urea content in the culture medium. The utilization rate of amino acids was obtained by dividing the concentration of amino acids remaining in the medium by the initial amino acid concentration. The urea and amino acid contents were determined by high-performance liquid chromatography (HPLC) using a Shimadzu LC-20A instrument (Shimadzu, Kyoto, Japan) equipped with a C18 column (4.6 × 150 mm × 3 μm; Thermo Scientific, CA, United States). Sample treatment and urea detection were carried out as described previously (Zhang et al., 2018a); urea was detected using a fluorescence detector at an excitation wavelength of 213 nm and an emission wavelength of 308 nm. The amino acid content was determined as described previously (Cigic et al., 2008); amino acids were detected using an ultraviolet detector. Primary and secondary amino acids were detected at 338 nm and 262 nm, respectively.

### Real-Time Quantitative PCR

Recombinant strains were cultured in YPD medium to the mid-log phase, cells were collected and washed briefly by diethylpyrocarbonate-treated water (without RNase), and RNA was extracted by the hot phenol method (Scholes and Lewis, 2020). A PrimeScript RT reagent Kit (TaKaRa, Dalian, China) was used for cDNA transcription. SYBR Premix Ex Taq II (TaKaRa) was used to perform qPCR following the manufacturer’s protocol on a LightCycler 480 II Real-time PCR instrument (Roche Applied Science, Mannheim, Germany). *ACT1* served as a housekeeping gene, and S288C served as a control. The 2^−ΔΔCt^ method (Livak and Schmittgen, 2001) was used to determine the relative expression levels of genes. Primers used for real-time quantitative PCR (RT-qPCR) are listed in Supplementary Table S4.

### Chromatin Immunoprecipitation qPCR

*S. cerevisiae* cells were activated in 10 ml YPD liquid medium then transferred to 50 ml YPD liquid medium to OD_600_ = 0.1. Cells were cultured at 30°C and 220 rpm to OD_600_ = 0.6–0.8. ChIP was performed as described in previous study (Tam and van Werven, 2020). Formaldehyde (37%) was added and incubated at room temperature for 15 min for crosslinking, and glycine was added to the final concentration of 100 mM to terminate crosslinking. Cells were washed with ice-cold TBS buffer and fragmented with glass beads, and FA lysis buffer (50 mM HEPES, pH 7.5, 150 mM NaCl, 1 mM EDTA, 1% Triton X-100, 0.1% sodium deoxycholate, 0.1% SDS) containing protease inhibitor (Roche) was added. Chromatin was fragmented using a Bioruptor Pico instrument (Diagenode, Belgium) and the 30′ on 30′ off program, yielding chromatin fragments 200–500 bp in size after 12 cycles of operation. Immunomagnetic beads were incubated with chromatin for 2 h at 4°C, then incubated with the H3K14Ac/panH4Ac antibody at room temperature for 30 min for immunoprecipitation. The immunomagnetic beads were washed seven times with FA lysis buffer, high-salt FA buffer, and FA buffer with lithium. Crosslinking of protein and DNA was terminated using stop buffer, and DNA was recovered by the ethanol precipitation method. qPCR was performed to quantify the recovered DNA using a SYBR Premix Ex Taq II kit (TaKaRa) and a LightCycler 480 II Real-time PCR instrument (Roche Applied Science) following the manufacturer’s instructions. Non-immunoprecipitated samples (input) were used to correct the data, and IgG antibody was used as a negative control. Enrichment results were calculated as described previously (Pascual-Ahuir and Proft, 2012). All primers used for chromatin immunoprecipitation qPCR (ChIP-qPCR) are listed in Supplementary Table S5.

### Statistical Analyses

Data in figure legends were statistically analyzed using GraphPad Prism version 8.2.0 for Windows (Tam and van Werven, 2020).^2^ Statistical significance was determined by unpaired parametric two-tailed Welch’s *t*-test with 95% confidence, and corrected values of *p* are presented in figures.

## Results

### Comparative *in silico* Identification Reveals Numerous Nitrogen Preference-Related Transcripts

At 3 h, the expression of *PFK1*, which encodes a key enzyme of the glycolytic pathway, was significantly upregulated in preferred nitrogen sources and was higher in glutamine than arginase, indicating that the metabolism of glucose was significantly accelerated and affected by different nitrogen source conditions at this incubation time. The expression of genes encoding ribosomal proteins can be activated by the transcription factor Rap1, and the expression levels of these genes were extremely high in rapidly growing cells. In this experiment, there was no significant difference in the transcript levels of *RAP1*, indicating no significant difference in the growth of cells cultured under different nitrogen sources for 3 h. Among genes involved in nitrogen source metabolism, *TOR1*, a cytosolic amino acid sensing gene, showed no significant change in transcription. The transcriptional activator encoded by *GLN3* was more active under non-preferred nitrogen sources (such as urea) and repressed under preferred nitrogen sources. At 3 h, a moderate reduction in *GLN3* transcript levels under Gln conditions was detected. The transcriptional repressor encoding gene *GZF3* had enhanced transcript levels when glutamine and arginase were both used as nitrogen sources, relative to urea (Supplementary Figure S2). The above results indicated that part of the genes involved in nitrogen source metabolism had begun to respond to changes in nutrient conditions at 3 h, and although the transcription of glycolytic pathway genes appeared differentially affected, yeast cell growth was not obviously affected, so the nitrogen source impact time choice of 3 h was more reasonable.

**Figure 1:**
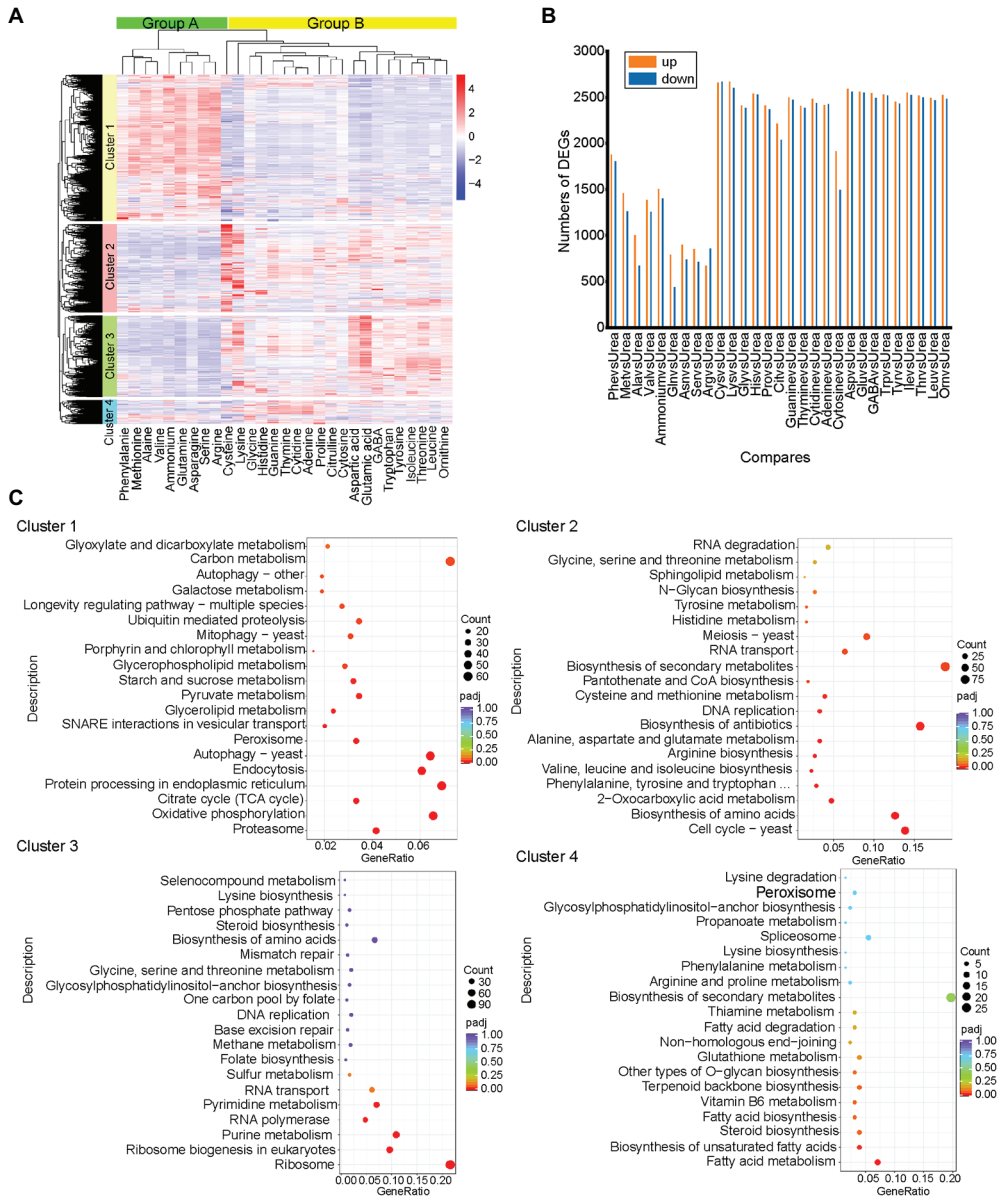
Overview of RNA-Seq data. **(A)** Clustering heatmap of all differentially expressed genes. Genes with similar transcription patterns were clustered into a cluster, resulting in four clusters. Samples were clustered to two groups. The thermogram shows the transcription of genes in different samples. The colors of squares represent values after normalization. Downregulated genes are highlighted in blue, and upregulated genes are highlighted in red. **(B)** Number of differentially expressed genes in each comparison group. Urea served as a control. Genes with an adjusted *value of p* <0.05 and |log_2_foldchange| >0.0 were considered differentially expressed. **(C)** KEGG analysis of the four clusters. KEGG pathway enrichment used padj <0.05 served as threshold criteria for significant enrichment. The 20 KEGG pathways with the most significant enrichment are shown in the scatter diagram.

To explore the response of *S. cerevisiae* S288C to different nitrogen sources at the transcriptional level, we compared RNA-Seq data from strain *S. cerevisiae* S288C exposed to 30 different nitrogen sources for 3 h. Only transcripts that exhibited an |log2foldchange| >0.0 and *p* < 0.05 were considered as significantly DEGs. Using these threshold criteria, a total of 6,446 DEGs with significant differences in expression were obtained (Figure 1A). Urea was chosen as the control since the catabolism and regulation of urea are widely understood, and other major regulatory systems, such as NCR and SPS mediated control, did not work when yeast was cultured on the medium with urea (Godard et al., 2007). Twenty-nine comparison groups compared with urea were obtained (Figure 1B). Hierarchical clustering analysis classified the DEGs into four different sized clusters, including cluster 1 (43%, 2,790 genes), cluster 2 (25%, 1,628 genes), cluster 3 (23%, 1,495 genes), and cluster 4 (7%, 440 genes; Figure 1B). In this study, the nitrogen sources that inhibits the utilization of non-preferred nitrogen source by S288C were defined as preferred nitrogen source (Glutamine, asparagine, aspartic acid, arginine, serine, alanine), and other nitrogen sources were defined as non-preferred nitrogen source (Godard et al., 2007). The nitrogen sources were clustered into two groups by hierarchical clustering analysis, in which group A included all preferred nitrogen sources except aspartic acid, including ammonium, asparagine, glutamine, methionine, phenylalanine, valine, alanine, serine, and arginine. Group B included other nitrogen sources (Figure 1A).

Most of the DEGs in cluster 1 showed an upregulated trend in group A and a downregulated trend in group B, while most of the DEGs in the other three clusters showed the opposite expression trend in the two groups. KEGG functional annotation of DEGs in the four clusters showed that the genes in cluster 1 were significantly enriched in carbon metabolism-related pathways, while the DEGs in cluster 2, cluster 3, and cluster 4 were significantly enriched in amino acid biosynthesis, purine and pyrimidine metabolism and fatty acid biosynthesis, respectively (Figure 1C). These results showed that *S. cerevisiae* cells undergo rapid growth in the presence of preferred nitrogen, whereas cells mainly carry out the synthesis and metabolism of secondary metabolites in the presence of the non-preferred nitrogen. Interestingly, we also obtained 2% (93) novel genes named novel. 1–93. Among them, only novel. 63 was annotated in GO, and its related biological processes mainly included various metabolic processes of cells (Supplementary Table S6). Additionally, novel. 83 was annotated as homologous recombination-related genes in KEGG (Supplementary Table S7). None of the other novel genes could be functionally annotated in the database. Information for all novel genes is listed in Supplementary Table S8.

### Changes in CRs Under the Most Preferred Nitrogen Source Conditions Are Consistent With Changes in NCR-Related Genes

**Figure 2:**
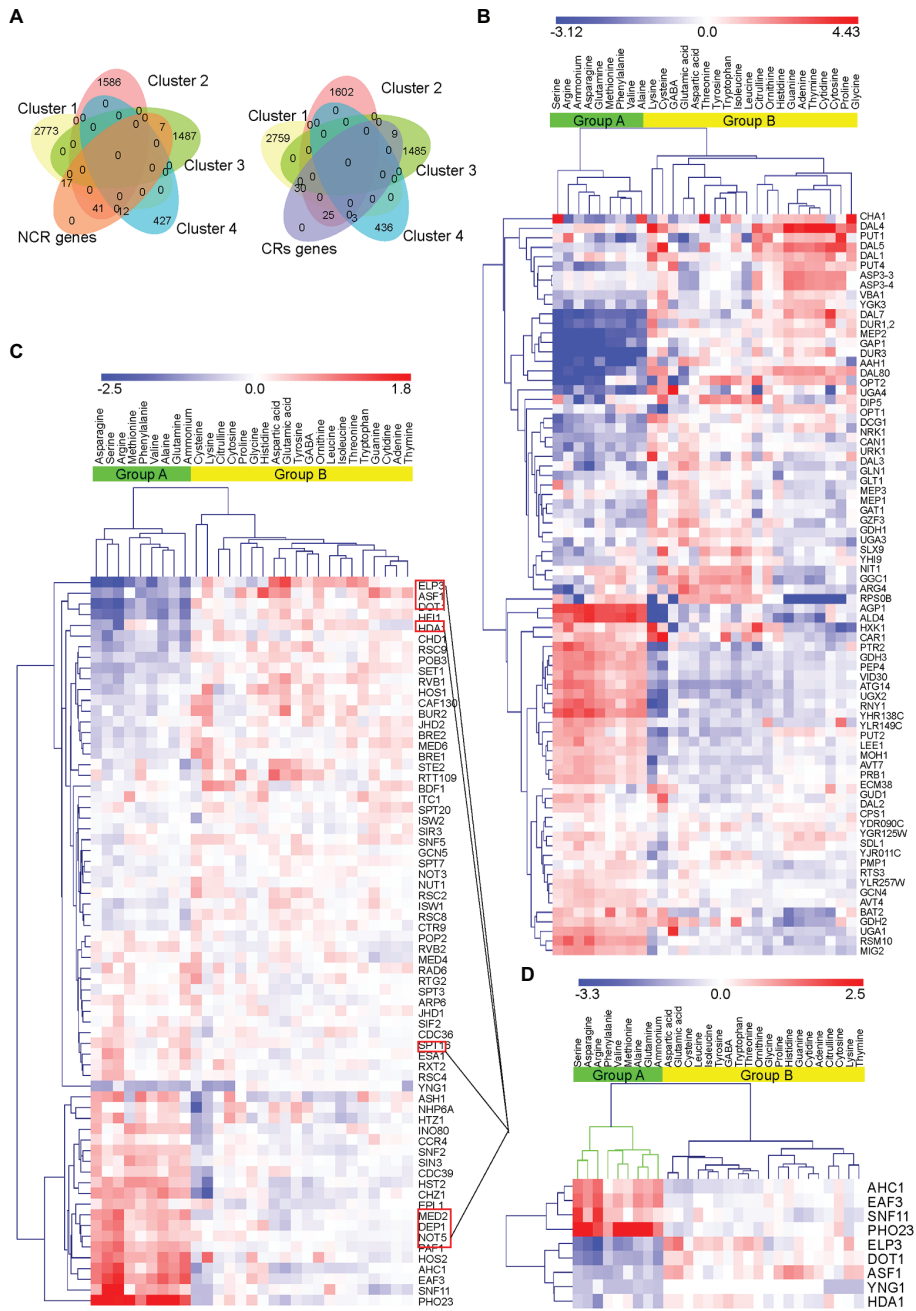
Venn diagrams and heatmaps of NCR target genes and CRs enrichment. **(A)** Venn diagrams of NCR and CRs in the four clusters. A total of 78 NCR genes and 67 CRs were obtained from the four clusters. **(B)** Hierarchical clustering of 78 NCR genes in response to changes in nitrogen sources based on normalized log2FoldChange. The green cluster (Group A) represents the preferred nitrogen sources of S288C obtained by clustering. Downregulated genes are highlighted in blue and upregulated genes are highlighted in red. **(C)** Hierarchical clustering of 67 CRs in response to changes in nitrogen sources based on normalized log_2_foldchange. Group A (green) obtained by clustering is consistent with NCR genes. Red square frames indicate nine CRs showing significant changes in transcription in the presence of various nitrogen sources. **(D)** Enrichment heatmap of the nine CRs showing the most significant differences in transcription in response to nitrogen sources among 67 CRs.

Based on previous studies on the effects of different preferred nitrogen sources on the nucleosome arrangement of *S. cerevisiae*, we speculate that the regulation of chromatin may be related to the preference of nitrogen source utilization of *S. cerevisiae*. Therefore, we compared the expression changes of NCR genes known to regulate nitrogen source preference under different nitrogen source conditions with the expression changes of CRs. According to previous findings, 78 NCR genes (Godard et al., 2007) and 67 CRs (Venters et al., 2011) were manually sorted from 6,446 transcripts to explore the association with nitrogen preference (Figure 2A).

According to the differential expression data of NCR genes, we found that the trend of expression change of NCR genes in group A was opposite to that in group B (Figure 2B). These results are consistent with previous studies (Godard et al., 2007), indicating that NCR genes are affected by different nitrogen sources. We also found that CR transcripts displayed similar patterns to NCR transcripts. The enrichment of CRs indicated that changes in these transcripts tended to be consistent when ammonium, asparagine, glutamine, methionine, phenylalanine, valine, alanine, serine, and arginine were used as nitrogen sources (Figure 2C). The aforementioned results were consistent with those of NCR target gene enrichment analysis in group A.

Based on our RNA-Seq data set, among the 67 CRs, the transcriptional changes (|log2foldchange|) of 9 CRs significantly higher than that of the other CRs in most comparison groups (Figure 2D), and these were selected for further analysis. Among them, increased expression of *EAF3*, *PHO23* (PHOsphate metabolism), *AHC1*, *YNG1* (Yeast iNG1 homolog), and *SNF11* (Sucrose Non-fermenting) was observed in the presence of preferred nitrogen sources, whereas reduced or similar expression patterns were revealed in the presence of non-preferred nitrogen sources. In contrast, expression levels of *ELP3* (ELongator Protein), *DOT1* (Disruptor Of Telomeric silencing), *HDA1* (Histone DeAcetylase) and *ASF1* (Anti-Silencing Function) were significantly reduced or unchanged in the presence of preferred nitrogen sources. These results suggested that these genes may have some effect on nitrogen metabolism in *S. cerevisiae* S288C.

### Overexpression of *AHC1*/*EAF3* Positively Influences Nitrogen Metabolism

**Figure 3:**
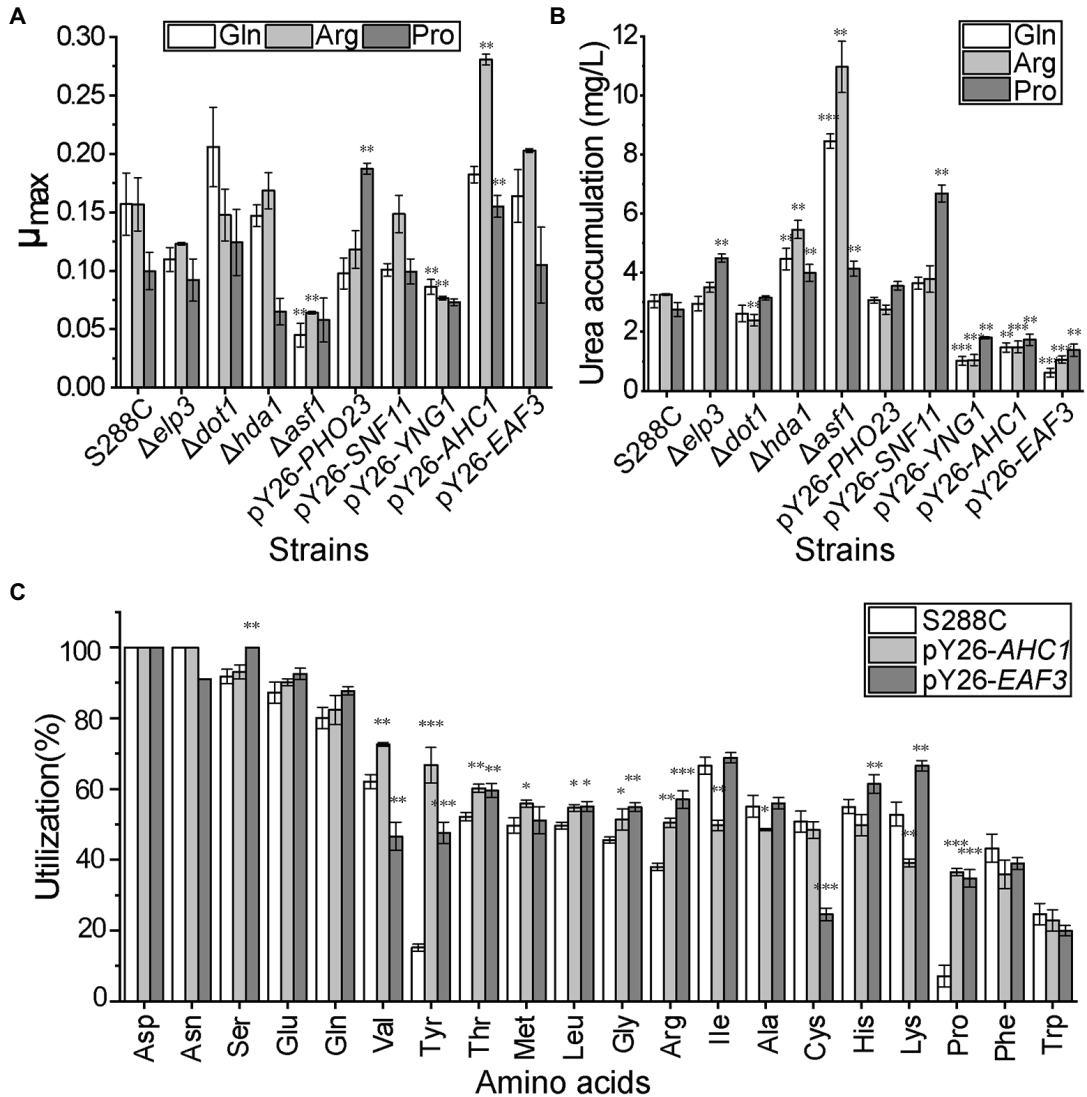
Physiological index analysis of CRs recombinant strains. **(A)** Growth status of nine CR recombinant strains in the presence of different nitrogen sources. **(B)** Urea accumulation in nine CR recombinant strains in the presence of different nitrogen sources. **(C)** Amino acid utilization rate of S288C-pY26-*AHC1* and S288C-pY26-*EAF3* in mixed medium containing the 20 common amino acids. Results are mean (M) ± standard deviation (SD) of three biological replicates. Statistical significance was determined by unpaired parametric two-tailed Welch’s *t*-test with 95% confidence (^*^p < 0.05, ^**^p < 0.005, ^***^p < 0.0005).

To further confirm that the expression of these genes could affect nitrogen usage, we speculated that overexpressing *EAF3*, *PHO23*, *AHC1*, *YNG1*, and *SNF11* and knocking out *ELP3*, *DOT1*, *HDA1*, and *ASF1* would increase the utilization of non-preferred nitrogen sources. Since glutamine is a common preferred nitrogen source of *S. cerevisiae*, arginine is the major precursor of urea and proline is a well-known non-preferred nitrogen source, they were used as sole nitrogen sources to verify the metabolic changes of recombinant strains under different nitrogen sources. The results showed that the growth of S288C-pY26-*AHC1* and S288C-pY26-*EAF3* was better than or close to that of S288C after 48 h fermentation. In the presence of three nitrogen sources, the μ_max_ of S288C-pY26-*AHC1* was 1.16, 1.79, and 1.55 times higher than that of S288C and the μ_max_ of S288C-pY26-*EAF3* was 1.04, 1.29, and 1.05 times of S288C, respectively (Figure 3A). Overexpression of *AHC1* significantly increased the growth rate of S288C when arginine and proline as the sole nitrogen source. However, the growth of S288C-Δ*asf1* and S288C-pY26-*YNG1* was significantly worse than that of S288C. These findings indicated that the modification of *ASF1* and *YNG1* affected yeast growth, so that S288C-Δ*asf1*, and S288C-pY26-*YNG1* were excluded from subsequent studies.

Urea is both a non-preferred nitrogen source for *S. cerevisiae* and the main precursor of the harmful nitrogen metabolite ethyl carbamate, the accumulation of urea is one of the key indicators to detect changes in nitrogen metabolism. The urea accumulation of S288C-pY26-*AHC1* and S288C-pY26-*EAF3* was significantly lower than that in the original strain. In the presence of three nitrogen sources, urea accumulation by S288C-pY26-*AHC1* for 48 h was 51.55, 54.50, and 37.21% lower than that of S288C (Figure 3B). Meanwhile, the urea accumulation of S288C-pY26-*EAF3* was reduced by 79.73, 67.49, and 50.04% compared with S288C, respectively (Figure 3B). However, the urea contents of S288C-Δ*dot1*, S288C-Δ*elp3*, S288C-Δ*hda1*, S288C-pY26-*PHO23*, and S288C-pY26-*SNF11* were similar to or significantly higher than that of S288C. Thus, subsequent studies excluded the aforementioned strains and focused on S288C-pY26-*AHC1* and S288C-pY26-*EAF3* with better urea reduction.

The utilization of the 20 common amino acids by S288C-pY26-*AHC1* and S288C-pY26-*EAF3* recombinant strains was then examined. As expected, utilization of non-preferred nitrogen sources including leucine, glycine, proline, and tyrosine and the preferred nitrogen source arginine was increased in both recombinant strains (Figure 3C). In addition, utilization of valine and methionine was increased in S288C-pY26-*AHC1*, while threonine, lysine, histidine, and serine utilization was increased in S288C-pY26-*EAF3*. These results confirmed the hypothesis that overexpression of *AHC1* and *EAF3* was indeed conducive to the utilization of non-preferred nitrogen sources.

### Overexpression of *AHC1*/*EAF3* Affects the Expression of Multiple Key Nitrogen Metabolism-Related Genes

**Figure 4:**
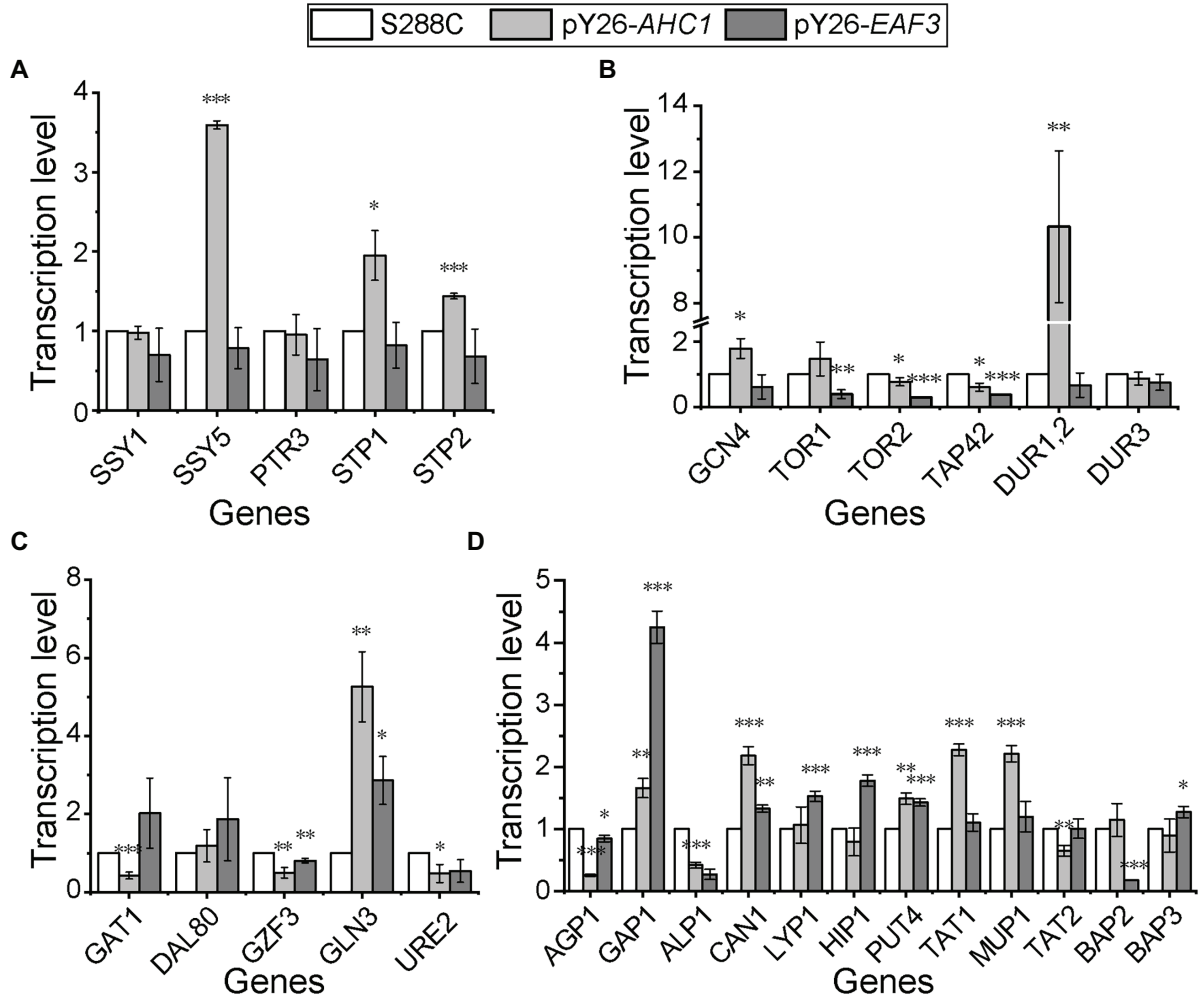
Relative transcription levels of key genes for nitrogen metabolism. **(A)** Relative transcription levels of SPS sensor system-related genes. **(B)** Relative transcription levels of genes related to the general amino acid control (GAAC) pathway, the TOR pathway, and urea metabolism. **(C)** Relative transcription levels of NCR genes. **(D)** Relative transcription levels of amino acid transporters. Genes with a transcriptional fold change greater or less than 1 were considered upregulated or downregulated, respectively. Transcription levels were normalized against the housekeeping gene *ACT1* and S288C as controls. Results are mean (M) ± standard deviation (SD) of three biological replicates. Statistical significance was determined by unpaired parametric two-tailed Welch’s *t*-test with 95% confidence (^*^p < 0.05, ^**^p < 0.005, ^***^p < 0.0005).

In order to detect whether the inhibition of the expression of genes related to the utilization of non-preferred nitrogen sources is weakened or relieved in the presence of preferred nitrogen sources by overexpression of *AHC1* and *EAF3*, qPCR was performed to measure their transcription levels under rich medium conditions. The results showed that most Ssy1-Ptr3-Ssy5 (SPS) signaling sensor system genes were upregulated in the *AHC1* overexpression strain, except for *SSY1* (Sulfonylurea Sensitive on YPD). In contrast, most genes of the SPS sensor system showed no significant changes in the *EAF3* overexpression strain (Figure 4A). Key genes of the TOR pathway all showed significant downregulation in both recombinant strains (Figure 4B). Urea carboxylase-encoding gene *DUR1,2* and *GCN4* (General Control Non-derepressible), a key gene of the GAAC pathway, were most strongly upregulated in the *AHC1* overexpression strain, but they were not significantly altered in the *EAF3* overexpression strain (Figure 4B). Furthermore, the results showed that the NCR repressors *GZF3* and *URE2* (UREidosuccinate transport) were significantly downregulated in both recombinant strains, whereas the NCR activator *GLN3* was upregulated. Another NCR activator was upregulated in the *EAF3* overexpression strain but was significantly downregulated in the *AHC1* overexpression strain (Figure 4C). In addition, overexpression of *AHC1* upregulated the expression of non-preferred nitrogen transporter-coding genes, including *PUT4* (Proline Utilization), *TAT1* (Tyrosine and tryptophan Amino acid Transporter), and *MUP1* (Methionine UPtake). In contrast, overexpression of *EAF3* upregulated the expression of non-preferred nitrogen transporter-encoding genes, including *LYP1* (Lysine-specific Permease), *HIP1* (Histidine Permease), *PUT4* and *BAP3* (Branched-chain Amino acid Permease). Moreover, the general amino acid transporter-encoding gene *GAP1* was upregulated in both overexpressing strains (Figure 4D).

### Overexpression of *AHC1*/*EAF3* Stimulates Histone Acetylation of Their Target Genes

**Figure 5:**
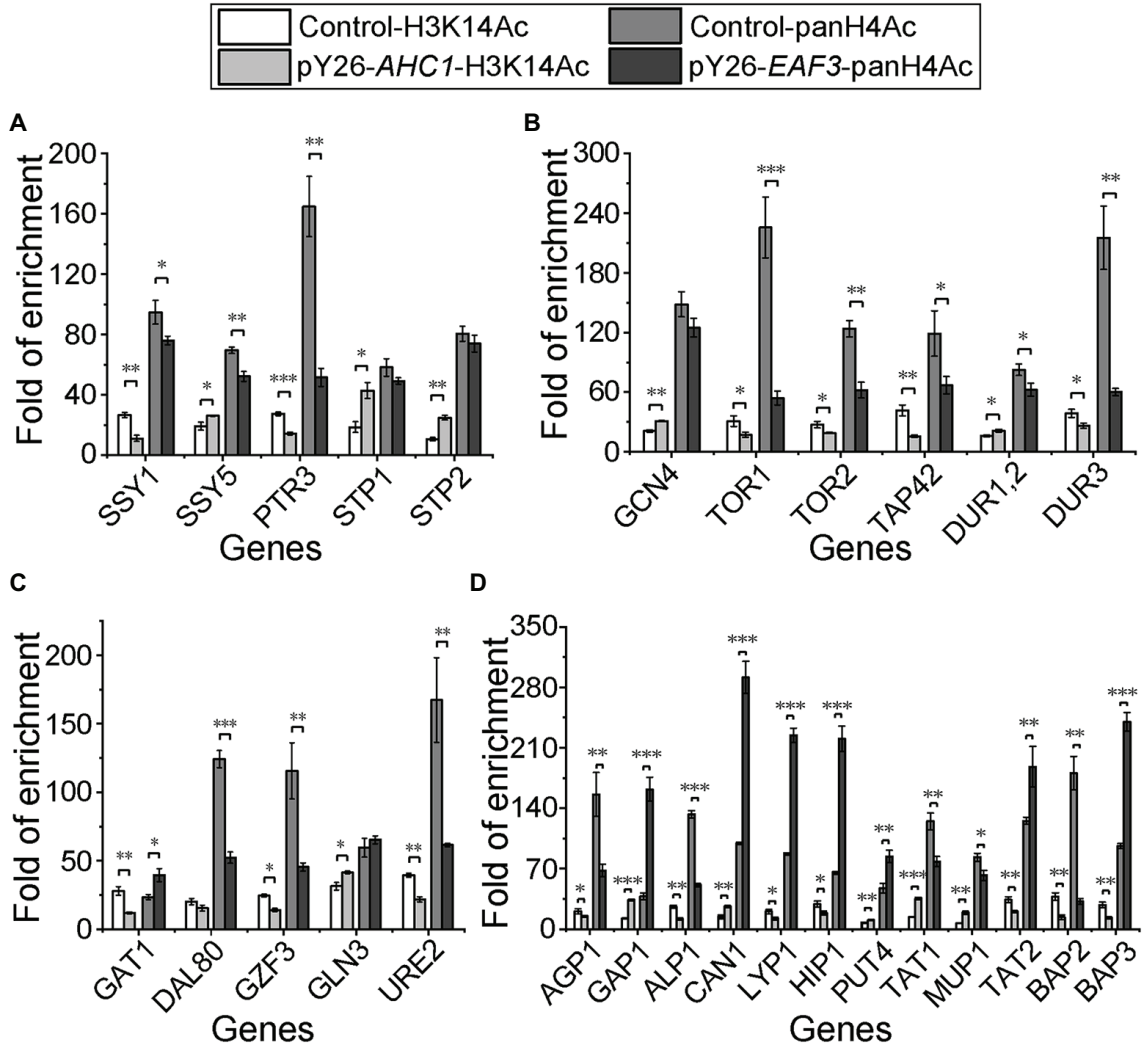
Histone acetylation levels of key nitrogen metabolism genes in *AHC1*/*EAF3* overexpression strains. **(A)** Histone acetylation levels of SPS sensor system-related genes. **(B)** Histone acetylation levels of genes related to the general amino acid control (GAAC) pathway, the TOR pathway, and urea metabolism. **(C)** Histone acetylation levels of NCR genes. **(D)** Histone acetylation levels of amino acid transporters. S288C served as a control. Signals were normalized against IgG, and results are mean (M) ± standard deviation (SD) of three biological replicates. Statistical significance was determined by unpaired parametric two-tailed Welch’s *t*-test with 95% confidence (^*^p < 0.05, ^**^p < 0.005, ^***^p < 0.0005).

Given that Ahc1p and Eaf3p are components of histone acetyltransferases (Grant et al., 1999; Sathianathan et al., 2016), we speculate that the upregulation of non-preferred nitrogen source utilization related genes may be due to the increased histone acetylation level on their promoters caused by the overexpression of *AHC1* and *EAF3*. Since the independent acetylation function of Ahc1p and Eaf3p has not been reported, we took the acetylation sites H3K14 and H4 of ADA and NuA4 complex as detection targets and detected the acetylation level of upregulated gene promoter region by ChIP-qPCR with H3K14Ac/panH4Ac antibodies. The results showed that H3K14 histone acetylation levels in the promoters of genes upregulated by *AHC1* overexpression were all significantly increased (Figure 5). Similarly, H4 histone acetylation levels were also significantly increased in the promoters of genes upregulated by *EAF3* overexpression (Figure 5). Histone acetylation levels at corresponding sites of genes not activated by *AHC1*/*EAF3* overexpression were measured at the same time. The results were in line with expectations, and histone acetylation levels at the corresponding sites were not increased (Figure 5). These results suggested that upregulation of target genes by *AHC1*/*EAF3* overexpression might depend on their functional interactions with histone acetyltransferase complexes.

## Discussion

Nitrogen sources are essential nutrients for the metabolism and growth of *S. cerevisiae* (Kessi-Perez et al., 2020). It is of great significance to explore the regulation of nitrogen metabolism in this model eukaryote. Previous ChIP (Ronsmans et al., 2019) and transcriptomic (Cubillos et al., 2017) analyses explored the nitrogen metabolism regulatory network of *S. cerevisiae*, although many details remain unresolved. To better understand nitrogen metabolism in this organism, we studied the effects of 30 nitrogen sources compared to 21 nitrogen sources in a previous study (Godard et al., 2007). We found that Ahc1p and Eaf3p regulated nitrogen metabolism, and we discovered 93 unannotated novel genes related to nitrogen metabolism. Future annotation of these genes may contribute significantly to further elucidating the nitrogen metabolism network in *S. cerevisiae*.

In contrast with the findings of a previous study (Godard et al., 2007), glutamate and aspartic acid were not clustered into the same group as other preferred nitrogen sources according to the clustering DEGs. This may be because the instantaneous changes in genes following the short-term impact of nitrogen sources on yeast cells differ from those caused by long-term culture (Abdul-Rahman et al., 2021). In addition, although glutamate supports the rapid growth of yeast cells, it cannot inhibit the utilization of non-preferred nitrogen sources in the S288C strain (Magasanik and Kaiser, 2002). A recent study revealed that ~12% of gene expression differences in the *S. cerevisiae* genome are positively correlated with chromatin accessibility differences under different nitrogen source conditions (Villarroel et al., 2021), proving the correlation between chromatin regulation and nitrogen metabolism. In this study, we found that the expression of CRs was significantly different under different nitrogen sources at the transcriptional level, further proving the critical role of chromatin regulation in nitrogen metabolism, verifying our previous research (Zhang et al., 2016).

Further functional analysis revealed that overexpression of *AHC1*/*EAF3* among CRs altered the expression of most nitrogen metabolism-related genes in a way that was conducive to using non-preferred nitrogen sources. Previous studies have shown that NCR transcription factors were regulated by the conserved TORC1 pathway (Molinet et al., 2019), but this regulation was partial (Fayyad-Kazan et al., 2016). Our current results showed that overexpression of *AHC1*/*EAF3* upregulated the transcription activators *GLN3* (*GLN3* and *GAT1* were upregulated in the *EAF3* overexpression strain) and downregulated the transcription inhibitors *GZF3* and *URE2* to relieve the inhibition of non-preferred nitrogen source-related genes (Springael and Penninckx, 2003; Tate et al., 2018), which may be one of the reasons for upregulation of the expression of the non-preferred nitrogen source transporter and urea metabolism-related gene *DUR1,2* (Wu et al., 2016). These results suggested that Ahc1p and Eaf3p may be the key regulators of NCR, other than TORC1. Additionally, overexpression of *AHC1* also affected the SPS sensor system and the GAAC pathway, which indicated the global regulation of nitrogen metabolism by Ahc1p. Furthermore, inhibition of TORC1 (*TOR1/2* and *TAP42*) by *AHC1*/*EAF3* overexpression might be due to the feedback of downstream regulatory factors (Varlakhanova et al., 2018) caused by a shift from the expression of intracellular nitrogen metabolism-related genes toward non-preferred nitrogen source utilization.

Further histone acetylation level detection showed that the upregulation of the target genes was due to the increase of histone acetylation level at their promoters, suggesting that Ahc1p and Eaf3p regulate non-preferred nitrogen-related genes through the function of their histone acetyltransferase complex. It should be noted that the histone acetylation level of *SSY5* promoter was only slightly upregulated, but its expression level was significantly upregulated. Since *SSY5* is regulated by Gcn4p, the upregulation of *GCN4* may be one of the reasons for the significant upregulation of *SSY5*.

**Figure 6:**
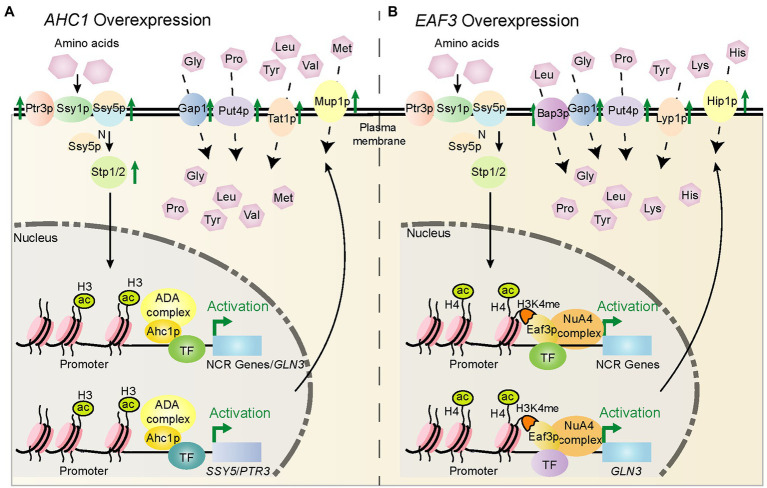
Schematic diagram of the potential mechanism by which *AHC1*/*EAF3* overexpression regulates key nitrogen metabolism genes. **(A)** Potential mechanism by which *AHC1* overexpression regulates nitrogen metabolism. Ahc1p may activate gene expression together with other transcription factors, and its effects are enhanced by overexpression of *AHC1*. Ahc1p depends on the ADA complex for its function to acetylate histone H3K14 sites on the promoters of target genes. This loosens nucleosomes and promotes the binding of Gln3p or other transcription factors, thereby activating the expression of target genes, resulting in upregulation of target gene expression, and eventually increased utilization of non-preferred nitrogen sources. **(B)** Potential mechanism by which *EAF3* overexpression regulates nitrogen metabolism. Eaf3p may act in a similar manner to Ahc1p. However, Eaf3p recognizes H3K4me sites on target gene promoters through its N-terminal chromodomain and localizes the NuA4 histone acetyltransferase complex to target gene promoters.

A previous study showed that the Spt-Ada-Gcn5-acetyltransferase (SAGA) complex (Wang et al., 2020) component Ada1p contributed to the transcription of multiple Gln3p- and Gat1p-dependent genes (Soussi-Boudekou and Andre, 1999). Ada1p was incorrectly identified as a component of the ADA complex in this previous study (Horiuchi et al., 1997). Similar to Ada1p, we found that the Ahc1p subunit of ADA complex (Helmlinger et al., 2021) and the Eaf3p subunit of NuA4 complex (Sathianathan et al., 2016) had positive regulatory effects on genes related to non-preferred nitrogen source utilization, among which the influence range of Ahc1p was more comprehensive. Therefore, we speculate that Ahc1p may act as a transcriptional coactivator to assist Gln3p or other transcription factors in regulating the expression of nitrogen metabolism-related genes. Besides, the Ahc1p contains a putative zinc finger DNA binding domain (Culbertson and Shogren-Knaak, 2021), which may be the structural basis for its regulatory function. In addition, as an acetylation module-relate subunit of ADA complex, the role of Ahc1p in nitrogen metabolism may depend on the acetylation function of its complex (Lee et al., 2011). Unlike Ahc1p, Eaf3p does not belong to the acetylation core of the NuA4 complex (Reid et al., 2004), which recognizes the H3K4me through its N-terminal chromodomain to locate NuA4 to histone H4 (Okuda and Nishimura, 2020). We speculate that Eaf3p may regulate the target gene by increasing the localization of NuA4 on the target gene promoter. Alternatively, it is a possibility that Eaf3p functions as a transcriptional coactivator of Gat1p and Gln3p or other transcription factors to co-regulate the expression of genes involved in the utilization of non-preferred nitrogen sources. The possible regulatory mechanisms are shown in Figure 6.

In this study, by analyzing changes in gene expression levels under different nitrogen sources, we revealed that chromatin regulator Ahc1p globally regulated nitrogen metabolism in *S. cerevisiae* S288C by co-regulating the expression of NCR, the SPS sensing system, the TOR pathway, and amino acid transporter genes. In addition, the chromatin regulatory Eaf3p regulated nitrogen metabolism at multiple levels by co-regulating NCR, the TOR pathway, and amino acid transporters. The regulation of nitrogen metabolism by Ahc1p and Eaf3p was dependent on their histone acetyltransferase complexes ADA and NuA4, which shifted nitrogen metabolism in the direction of non-preferred nitrogen source utilization. These results illuminated the regulatory mechanism of NCR and broadened our understanding of the nitrogen metabolism network of *S. cerevisiae*. The findings will assist future studies on the regulatory mechanisms by which Ahc1p and Eaf3p influence nitrogen metabolism.

## Data Availability Statement

The datasets presented in this study can be found in online repositories. The names of the repository/repositories and accession number(s) can be found in the article/Supplementary Material.

## Author Contributions

YC: conceptualization of study, molecular biology, cultivation, sample preparation, analysis, and writing. WZ: transcriptomics. WenM and WeiM: data analysis. JZ: reviewing and editing. All authors contributed to the article and approved the submitted version.

## Funding

This work was supported by the Foundation for Innovative Research Groups of the National Natural Science Foundation of China (32021005) and Tianjin Synthetic Biotechnology Innovation Capacity Improvement Project (TSBICIP-KJGG-004; National Technology Innovation Center of Synthetic Biology).

## Conflict of Interest

The authors declare that the research was conducted in the absence of any commercial or financial relationships that could be construed as a potential conflict of interest.

## Publisher’s Note

All claims expressed in this article are solely those of the authors and do not necessarily represent those of their affiliated organizations, or those of the publisher, the editors and the reviewers. Any product that may be evaluated in this article, or claim that may be made by its manufacturer, is not guaranteed or endorsed by the publisher.

## Supplementary Material

The Supplementary Material for this article can be found online at: https://www.frontiersin.org/articles/10.3389/fmicb.2022.883934/full#supplementary-material

## Footnotes

^1^ http://www.genome.jp/kegg/

^2^ www.graphpad.com
