# Supplementary material for: Chromatin Regulators Ahc1p and Eaf3p Positively Influence Nitrogen Metabolism in Saccharomyces cerevisiae
Source: Front Microbiol. 2022 May 10;13:883934. doi: 10.3389/fmicb.2022.883934 (PMC9127870; doi:10.3389/fmicb.2022.883934)
Supplement: Supplementary file 1 [file Table_1.DOCX]

Supplementary Material

## Supplementary Tables

### Table S1 List of strains used in this study.

| **Strains** | **Genotype** | **Source** |
| --- | --- | --- |
| S288C | *MATα SUC2 gal2 mal mel flo 1 flo8-1 hap1 ho bio1 bio6* | (Daran-Lapujade et al., 2003) |
| S288C-Δ*ura3* | S288C, *ura3*Δ | This study |
| S288C-Δ*elp3* | S288C-Δ*ura3*, *elp3*Δ | This study |
| S288C-Δ*dot1* | S288C-Δ*ura3*, *dot1*Δ | This study |
| S288C-Δ*hda1* | S288C-Δ*ura3*, *hda1*Δ | This study |
| S288C-Δ*asf1* | S288C-Δ*ura3,* *asf1*Δ | This study |
| S288C- pY26-*PHO23* | S288C-Δ*ura3*, pY26-*PHO23* | This study |
| S288C- pY26-*SNF11* | S288C-Δ*ura3*, pY26-*SNF11* | This study |
| S288C- pY26-*YNG1* | S288C-Δ*ura3*, pY26-*YNG1* | This study |
| S288C- pY26-*AHC1* | S288C-Δ*ura3*, pY26-*AHC1* | This study |
| S288C- pY26-*EAF3* | S288C-Δ*ura3*, pY26-*EAF3* | This study |

### Table S2 List of plasmids used in this study.

| **Plasmids** | **Genotype** | **Source** |
| --- | --- | --- |
| pRS426-Cas9-sgRNA | Ampr *URA3 P*TEF1*-Cas9*-TCYC1-sgRNA | Lab stored |
| pY26-TEF-GDP | *URA3*, 2μ, Ampr | (Li et al., 2008) |
| pY26-*AHC1* | pY26, P_TEF_*-AHC1-*T_ADH1_ | This study |
| pY26-*EAF3* | pY26, P_TEF_*-EAF3-*T_ADH1_ | This study |
| pY26-*PHO23* | pY26, P_TEF_*-PHO23-*T_ADH1_ | This study |
| pY26-*SNF11* | pY26, P_TEF_*-SNF11-*T_ADH1_ | This study |
| pY26-*YNG1* | pY26, P_TEF_*-YNG1-*T_ADH1_ | This study |

### Table S3 List of nitrogen sources used in this study.

| **No.** | **Nitrogen source** | **Concentration** |
| --- | --- | --- |
| 1 | Glycine | 10 mM |
| 2 | Alanine | 10 mM |
| 3 | Leucine | 10 mM |
| 4 | Isoleucine | 10 mM |
| 5 | Valine | 10 mM |
| 6 | Proline | 10 mM |
| 7 | Phenylalanine | 10 mM |
| 8 | Methionine | 10 mM |
| 9 | Tryptophan | 5 mM |
| 10 | Serine | 10 mM |
| 11 | Glutamine | 10 mM |
| 12 | Threonine | 10 mM |
| 13 | Cysteine | 10 mM |
| 14 | Asparagine | 10 mM |
| 15 | Tyrosine | 10 mM |
| 16 | Aspartic acid | 10 mM |
| 17 | Glutamic acid | 10 mM |
| 18 | Lysine | 10 mM |
| 19 | Arginine | 10 mM |
| 20 | Histidine | 10 mM |
| 21 | Ammonium sulfate | 10 mM |
| 22 | Urea | 10 mM |
| 23 | γ-aminobutyric acid (GABA) | 10 mM |
| 24 | Ornithine | 10 mM |
| 25 | Citrulline | 10 mM |
| 26 | Cytosine | 10 mM |
| 27 | Cytidine | 10 mM |
| 28 | Guanine | 10 mM |
| 29 | Adenine | 10 mM |
| 30 | Thymine | 10 mM |

### Table S4 List of RT-qPCR primers used in this study.

| **Primer name** | **Primer sequence (5'-3')** |
| --- | --- |
| GLN3-F | TCAACATGACGCCCAGCAACTC |
| GLN3-R | CGGGAACAGTGAGGATTTGGAGAC |
| GAT1-F | AATCATTGCGTCCGACCACAGG |
| GAT1-R | GGTAGCTGCACCGGCACTAAC |
| GZF3-F | GACCCAGCCCCTTTATCTTTCCAC |
| GZF3-R | AACGGTATGAAATGAGGCGGTGTC |
| DAL80-F | TGCTGCTGGAGTGGATGATTGTG |
| DAL80-R | GGTGTCCGTCTTCAAGCTGATAGG |
| URE2-F | CATGCGCCAATGATTGGACAAGC |
| URE2-R | CTACGTTCAGCCAAGGCCATCTC |
| SSY1-F | CCAAAAGGCCGATTTTTAGCTA |
| SSY1-R | TCCTTGGATTTATAGCTTCCCC |
| PTR3-F | TTCAATTCATCTCCACTGGTCA |
| PTR3-R | CACTTGTGAATCTTGCATCCAA |
| SSY5-F | GCAGTTTCAGAGAATCCACTTG |
| SSY5-R | TCACTACCAGTCTGAGGGAATA |
| STP1-F | AGCTCTCCATTAAGTCCATCAG |
| STP1-R | CCTCTAATCCTGAAAGTAGCGT |
| STP2-F | TGGATTCTCCAGCATTATCCTC |
| STP2-R | AACGATTGCTCTGAATCCAATG |
| GCN4-F | CCGCAAACAGCGTTCGATTC |
| GCN4-R | CTGGCGGCTTCAGTGTTTCTA |
| TOR1-F | GAAAGTGGAAAGTTTCGAAGCT |
| TOR1-R | TAACTTTCATTGATCAACGCGG |
| TOR2-F | GCTGGCTGCTTCCTCTGGTTATG |
| TOR2-R | ACGAACAGTTCCACGCCTGATATG |
| TAP42-F | TCTTGAGGAACTTCACGCTTGTCG |
| TAP42-R | TTCATCTGGTGCTTGCTCTGGTTC |
| DUR1,2-F | ATGGTGCGGTGGTGGTATTGTAAG |
| DUR1,2-R | CTTGTCCTCTGCTCTTCTCTTGCC |
| DUR3-F | TTCATGGCCGTCACATCTGCTATG |
| DUR3-R | TTCTTACCGCTTGCACGAGGATC |
| AGP1-F | GCTGTTGCATCCCACGGTGTC |
| AGP1-R | CGAGCACTGGAGTAGAAGGAGGAG |
| GAP1-F | CATTGAAGCACCACTTGAAGAA |
| GAP1-R | CTGTTCTTAGTGCAGTACCTGA |
| ALP1-F | CAATCGTTAGGAGAGATGGTCA |
| ALP1-R | CCACGACAGCCAGTACATATAT |
| CAN1-F | GAGCCGGTCACAACCCTCTT |
| CAN1-R | GTCGGGAAAGAGCGCAATGG |
| LYP1-F | GATGCCTTATGGTGCCTACTACGC |
| LYP1-R | TGAACGGACAGAACGCTTGGAAC |
| HIP1-F | ATTCACCTGGCATAAACCAAAC |
| HIP1-R | TCTTTACTCAGGTTGGTGTTGT |
| PUT4-F | CAGAATCAACAAATGGGGTGTT |
| PUT4-R | GGTGCTGATATTGGAAAACCAA |
| TAT1-F | GGTAATGGTCAGGTGCTGGGAAC |
| TAT1-R | CGCCTGCCGCTTGGATGATAC |
| TAT2-F | CTGGGTGGTTCATCTGTGGACAAC |
| TAT2-R | ACGATGGAGGGCAAGGCTTTAATG |
| MUP1-F | TTGATGTGCTTTGCCGGGCTATAC |
| MUP1-R | AGCAATAGAGGACACCGACAGGAG |
| BAP2-F | GCTGCGCTAATAATTGGTTACA |
| BAP2-R | TGAAATTTGCTGGTAAAGTCGG |
| BAP3-F | CAACAACAGCTTCACTAACTCC |
| BAP3-R | TTCTTCTAAACGTTGGCTTTCG |

### Table S5 List of ChIP-qPCR primers used in this study.

| **Primer name** | **Primer sequence (5'-3')** |
| --- | --- |
| P_GLN3_-F | TGGCAATGCTGAGAGAGTGGAAAG |
| P_GLN3_-R | TGTTATTGTGGGATGGGCTACTGC |
| P_GAT1_-F | GCCGACACATAGCAGAACGATGTG |
| P_GAT1_-R | GCAAGTGTGCGACTCATAGTGCTG |
| P_DUR1,2_-F | CGCTCACTTCTGAATATCAGGCTCT |
| P_DUR1,2_-R | TGGTGTATATCAGCTGCTCCACTGA |
| P_GCN4_-F | ACCAATTGCTATCATGTACCCGT |
| P_GCN4_-R | TGGCGAGTAAACCTGGATAATTTGA |
| P_SSY5_-F | AGTGAAATTGTAGAACGCCGCG |
| P_SSY5_-R | GGAGGATATCTAGTATGTTTATTCAACC |
| P_STP1_-F | GATGGAAATGAAGGTGCCCACTGAC |
| P_STP1_-R | CAGCAGGAGACAGTCAAGCGTTC |
| P_TAT1_-F | TCCCTGTTTTGTGGCATCTATTTCA |
| P_TAT1_-R | GCTACGGTGGTAAAGGCACC |
| P_PUT4_-F | GGGGTTTGTGTTCCTCTTCCTTTCC |
| P_PUT4_-R | GATGGTGTGATTCTTCGAAACAGC |
| P_MUP1_-F | TTCCTCCTACTTGGCGGTGAATTTC |
| P_MUP1_-R | TGAACAGCGGAAAAGTTTGTGTGAG |
| P_GAP1_-F | CGCTCTGGATGAGACATATAAAGATG |
| P_GAP1_-R | TGTTGCTGTCCTTGGTCTGTTCTT |
| P_CAN1_-F | CGAATCAGGGAATCCCTTTTTGCA |
| P_CAN1_-R | CTGAAATAAACTTTCGATTGACGACAG |
| P_BAP3_-F | TGCGTTTCCTTGTGGTAAATCGG |
| P_BAP3_-R | CGCCAATCCATCAATAGTAACCACG |
| P_HIP1_-F | GTGCTAGTTTGTCCATTGTTGTGTCA |
| P_HIP1_-R | ACTTGGGAAGGACAATGAATTGTA |
| P_LYP1_-F | GTCGATGATCACAATTGTTAGGTGACT |
| P_LYP1_-R | TGTGTAACTAGAGGAGGCCGG |

### Table S6 GO annotation of novel genes.

| Gene ID | GO ID | GO ontology | GO term |
| --- | --- | --- | --- |
| novel.63 | GO:0015074 | BP | DNA integration |
| novel.63 | GO:0008152 | BP | metabolic process |
| novel.63 | GO:0006139 | BP | nucleobase-containing compound metabolic process |
| novel.63 | GO:0006259 | BP | DNA metabolic process |
| novel.63 | GO:0006725 | BP | cellular aromatic compound metabolic process |
| novel.63 | GO:0006807 | BP | nitrogen compound metabolic process |
| novel.63 | GO:0008150 | BP | biological_process |
| novel.63 | GO:0009987 | BP | cellular process |
| novel.63 | GO:0034641 | BP | cellular nitrogen compound metabolic process |
| novel.63 | GO:0043170 | BP | macromolecule metabolic process |
| novel.63 | GO:0044237 | BP | cellular metabolic process |
| novel.63 | GO:0044238 | BP | primary metabolic process |
| novel.63 | GO:0044260 | BP | cellular macromolecule metabolic process |
| novel.63 | GO:0046483 | BP | heterocycle metabolic process |
| novel.63 | GO:0071704 | BP | organic substance metabolic process |
| novel.63 | GO:0090304 | BP | nucleic acid metabolic process |
| novel.63 | GO:1901360 | BP | organic cyclic compound metabolic process |
| novel.63 | GO:0005737 | CC | cytoplasm |
| novel.63 | GO:0005622 | CC | intracellular |
| novel.63 | GO:0005575 | CC | cellular_component |
| novel.63 | GO:0005623 | CC | cell |
| novel.63 | GO:0044424 | CC | intracellular part |
| novel.63 | GO:0044464 | CC | cell part |
| novel.63 | GO:0003723 | MF | RNA binding |
| novel.63 | GO:0003674 | MF | molecular_function |
| novel.63 | GO:0003676 | MF | nucleic acid binding |
| novel.63 | GO:0005488 | MF | binding |
| novel.63 | GO:0097159 | MF | organic cyclic compound binding |
| novel.63 | GO:1901363 | MF | heterocyclic compound binding |

### Table S7 KEGG annotation of novel genes.

| Gene ID | Pathway gene_ID | Pathway ID | Pathway name |
| --- | --- | --- | --- |
| novel.83 | YLR234W | sce03440 | Homologous recombination |

### Table S8 Novel genes predicted from transcriptome data.

| Gene id | Chromosome number | Starting coordinate | Termination coordinate | Gene strand | Gene length |
| --- | --- | --- | --- | --- | --- |
| novel.1 | NC_001133.9 | 151594 | 152152 | + | 559 |
| novel.2 | NC_001133.9 | 208184 | 218139 | - | 9956 |
| novel.3 | NC_001134.8 | 7884 | 9532 | + | 1649 |
| novel.4 | NC_001134.8 | 642212 | 642579 | + | 368 |
| novel.5 | NC_001134.8 | 107682 | 107931 | - | 250 |
| novel.6 | NC_001135.5 | 9464 | 9691 | + | 228 |
| novel.7 | NC_001135.5 | 78716 | 79055 | + | 340 |
| novel.8 | NC_001135.5 | 127993 | 128463 | - | 471 |
| novel.9 | NC_001135.5 | 294376 | 294629 | - | 254 |
| novel.10 | NC_001136.10 | 56350 | 56747 | + | 398 |
| novel.11 | NC_001136.10 | 1516628 | 1517124 | + | 497 |
| novel.12 | NC_001136.10 | 21234 | 22568 | - | 1335 |
| novel.13 | NC_001136.10 | 80639 | 83544 | - | 2906 |
| novel.14 | NC_001136.10 | 452825 | 453043 | - | 219 |
| novel.15 | NC_001136.10 | 620181 | 622086 | - | 1906 |
| novel.16 | NC_001136.10 | 694380 | 694699 | - | 320 |
| novel.17 | NC_001136.10 | 930650 | 931101 | - | 452 |
| novel.18 | NC_001137.3 | 7790 | 13360 | + | 5571 |
| novel.19 | NC_001137.3 | 248160 | 250019 | + | 1860 |
| novel.20 | NC_001137.3 | 287707 | 288434 | + | 728 |
| novel.21 | NC_001137.3 | 562887 | 565367 | + | 2481 |
| novel.22 | NC_001138.5 | 5417 | 6263 | + | 847 |
| novel.23 | NC_001138.5 | 110194 | 110607 | - | 414 |
| novel.24 | NC_001138.5 | 233940 | 234241 | - | 302 |
| novel.25 | NC_001138.5 | 260451 | 263968 | - | 3518 |
| novel.26 | NC_001139.9 | 627187 | 627597 | + | 411 |
| novel.27 | NC_001139.9 | 793255 | 794407 | + | 1153 |
| novel.28 | NC_001139.9 | 875204 | 876316 | + | 1113 |
| novel.29 | NC_001139.9 | 993525 | 994063 | + | 539 |
| novel.30 | NC_001139.9 | 16432 | 18597 | - | 2166 |
| novel.31 | NC_001139.9 | 114920 | 115478 | - | 559 |
| novel.32 | NC_001139.9 | 319880 | 321600 | - | 1721 |
| novel.33 | NC_001139.9 | 371287 | 372011 | - | 725 |
| novel.34 | NC_001139.9 | 421291 | 423090 | - | 1800 |
| novel.35 | NC_001139.9 | 994174 | 995428 | - | 1255 |
| novel.36 | NC_001140.6 | 10215 | 11884 | + | 1670 |
| novel.37 | NC_001140.6 | 48156 | 48605 | + | 450 |
| novel.38 | NC_001140.6 | 116340 | 117469 | + | 1130 |
| novel.39 | NC_001140.6 | 302270 | 302579 | + | 310 |
| novel.40 | NC_001140.6 | 516666 | 517531 | - | 866 |
| novel.41 | NC_001140.6 | 528787 | 537904 | - | 9118 |
| novel.42 | NC_001140.6 | 541129 | 541594 | - | 466 |
| novel.43 | NC_001141.2 | 32649 | 33378 | + | 730 |
| novel.44 | NC_001141.2 | 75784 | 76350 | + | 567 |
| novel.45 | NC_001141.2 | 195083 | 195370 | - | 288 |
| novel.46 | NC_001142.9 | 667390 | 667638 | - | 249 |
| novel.47 | NC_001142.9 | 669321 | 669521 | - | 201 |
| novel.48 | NC_001143.9 | 73994 | 74557 | + | 564 |
| novel.49 | NC_001143.9 | 513428 | 514791 | + | 1364 |
| novel.50 | NC_001143.9 | 18602 | 21209 | - | 2608 |
| novel.51 | NC_001143.9 | 201999 | 202983 | - | 985 |
| novel.52 | NC_001143.9 | 248598 | 248919 | - | 322 |
| novel.53 | NC_001143.9 | 364220 | 364773 | - | 554 |
| novel.54 | NC_001143.9 | 442991 | 443230 | - | 240 |
| novel.55 | NC_001143.9 | 513453 | 515052 | - | 1600 |
| novel.56 | NC_001143.9 | 660788 | 661441 | - | 654 |
| novel.57 | NC_001144.5 | 490878 | 491862 | + | 844 |
| novel.58 | NC_001144.5 | 796748 | 797099 | + | 352 |
| novel.59 | NC_001144.5 | 1062498 | 1062794 | + | 297 |
| novel.60 | NC_001144.5 | 733883 | 734587 | - | 577 |
| novel.61 | NC_001144.5 | 924901 | 925566 | - | 666 |
| novel.62 | NC_001144.5 | 928171 | 928623 | - | 453 |
| novel.63 | NC_001144.5 | 976257 | 982893 | - | 6557 |
| novel.64 | NC_001144.5 | 1052147 | 1053310 | - | 1164 |
| novel.65 | NC_001145.3 | 306429 | 307481 | + | 1053 |
| novel.66 | NC_001145.3 | 321263 | 321799 | + | 537 |
| novel.67 | NC_001145.3 | 378374 | 379173 | + | 758 |
| novel.68 | NC_001145.3 | 731333 | 732251 | + | 919 |
| novel.69 | NC_001145.3 | 31687 | 32331 | - | 645 |
| novel.70 | NC_001145.3 | 434189 | 434785 | - | 597 |
| novel.71 | NC_001145.3 | 480804 | 483013 | - | 2210 |
| novel.72 | NC_001145.3 | 503169 | 504890 | - | 1722 |
| novel.73 | NC_001145.3 | 659477 | 659735 | - | 259 |
| novel.74 | NC_001145.3 | 837009 | 837765 | - | 757 |
| novel.75 | NC_001145.3 | 919603 | 920079 | - | 477 |
| novel.76 | NC_001146.8 | 724376 | 726104 | + | 1729 |
| novel.77 | NC_001146.8 | 779332 | 779850 | + | 519 |
| novel.78 | NC_001146.8 | 394386 | 394683 | - | 298 |
| novel.79 | NC_001146.8 | 635512 | 635898 | - | 387 |
| novel.80 | NC_001146.8 | 663582 | 664242 | - | 661 |
| novel.81 | NC_001147.6 | 42644 | 43688 | - | 1045 |
| novel.82 | NC_001147.6 | 99493 | 99803 | - | 311 |
| novel.83 | NC_001147.6 | 461836 | 462765 | - | 930 |
| novel.84 | NC_001147.6 | 682395 | 683101 | - | 707 |
| novel.85 | NC_001147.6 | 908357 | 909174 | - | 818 |
| novel.86 | NC_001147.6 | 1010900 | 1011183 | - | 284 |
| novel.87 | NC_001147.6 | 1073379 | 1074196 | - | 818 |
| novel.88 | NC_001147.6 | 1079803 | 1080274 | - | 472 |
| novel.89 | NC_001148.4 | 347561 | 348383 | + | 823 |
| novel.90 | NC_001148.4 | 775837 | 777131 | + | 1295 |
| novel.91 | NC_001148.4 | 856378 | 856827 | + | 450 |
| novel.92 | NC_001148.4 | 99089 | 99483 | - | 395 |
| novel.93 | NC_001148.4 | 143399 | 143820 | - | 422 |

## Supplementary Figures


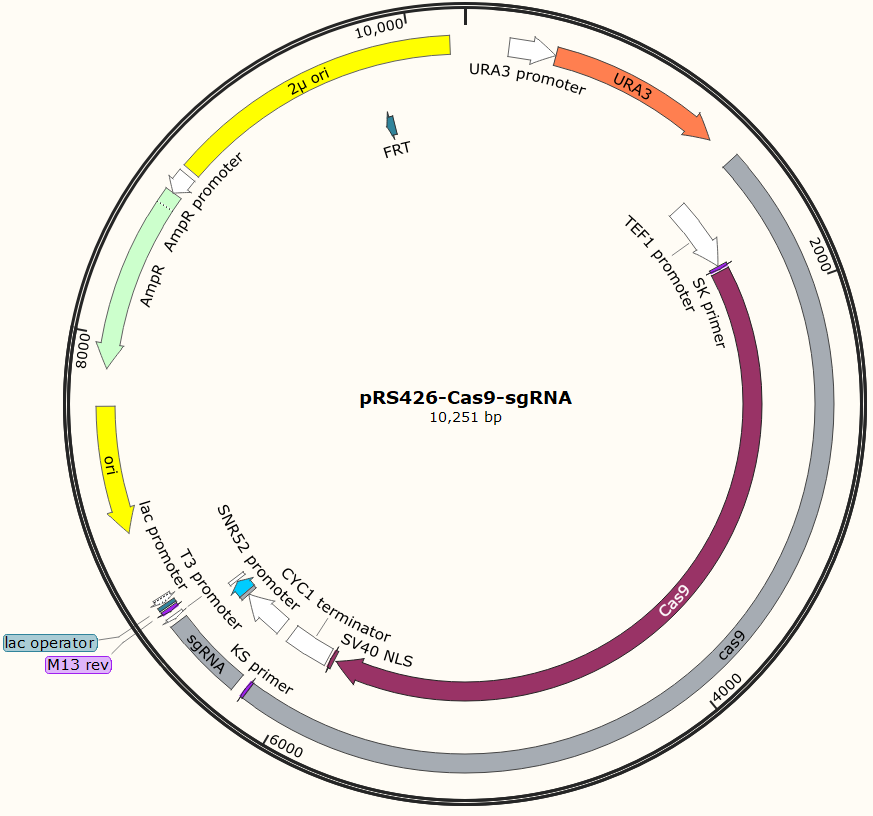


### Figure S1 The plasmid map of pRS426-Cas9-sgRNA.


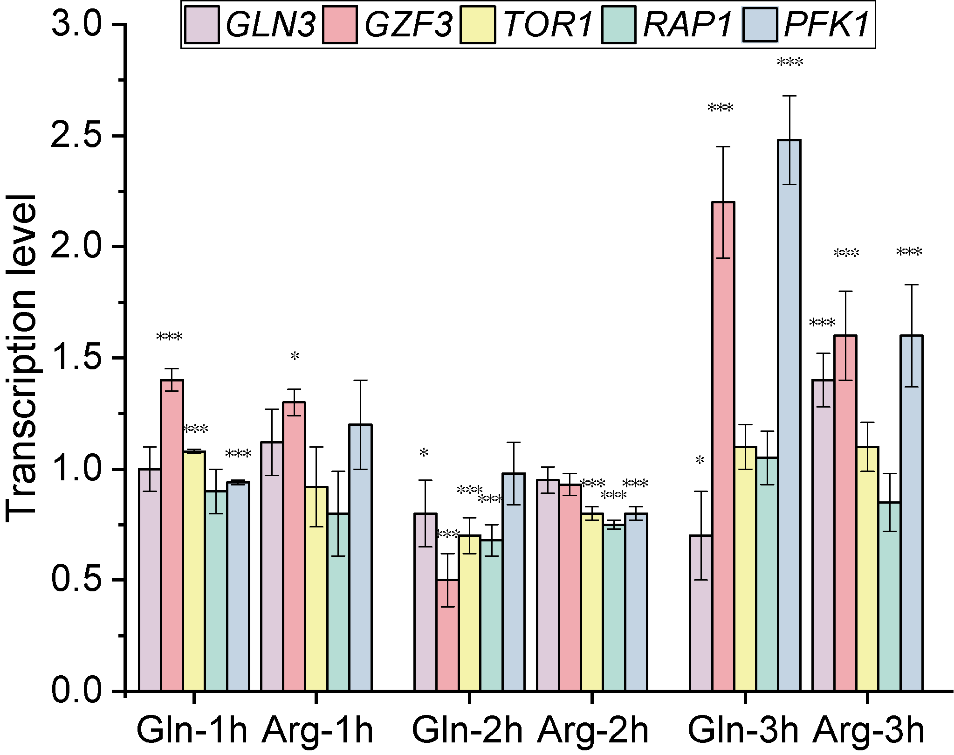


### Figure S2 Differences of nitrogen metabolism-related gene transcription levels of S288C under different nitrogen sources and different culture times.

Genes with a transcriptional fold change greater or less than 1 were considered upregulated or downregulated, respectively. Transcription levels were normalised against the housekeeping gene *ACT1* and S288C treated with urea as controls. Results are mean (M) ± standard deviation (SD) of three biological replicates. Statistical significance was determined by unpaired parametric two-tailed Welch’s t-test with 95% confidence (^*^*p* <0.05, ^**^*p* <0.005, ^***^*p* <0.0005).

Daran-Lapujade, P., Daran, J.M., Kotter, P., Petit, T., Piper, M.D., and Pronk, J.T. (2003). Comparative genotyping of the *Saccharomyces cerevisiae* laboratory strains S288C and CEN.PK113-7D using oligonucleotide microarrays. *FEMS Yeast Res* 4(3)**,** 259-269. doi: 10.1016/S1567-1356(03)00156-9.
